# Managing Infection Complications in the Setting of Chimeric Antigen Receptor T cell (CAR-T) Therapy

**DOI:** 10.46989/001c.115932

**Published:** 2024-04-29

**Authors:** Nausheen Ahmed, Olalekan Oluwole, Zahra Mahmoudjafari, Nahid Suleman, Joseph P McGuirk

**Affiliations:** 1 Hematologic Malignancies and Cellular Therapeutics University of Kansas Cancer Center; 2 Medicine, Hematology and Oncology Vanderbilt University Medical Center

**Keywords:** Infections, CD19 CAR-T, BCMA, Prevention, Vaccinations, Prophylaxis, Immunizations, lymphoma, myeloma, lymphoblastic leukemia, Chimeric antigen receptor

## Abstract

Chimeric antigen receptor T-cell (CAR T-cell) therapy has changed the paradigm of management of non-Hodgkin’s lymphoma (NHL) and Multiple Myeloma. Infection complications have emerged as a concern that can arise in the setting of therapy and lead to morbidity and mortality.

In this review, we classified infection complications into three categories, pre-infusion phase from the time pre- lymphodepletion (LD) up to day zero, early phase from day of infusion to day 30 post-infusion, and late phase after day 30 onwards. Infections arising in the pre-infusion phase are closely related to previous chemotherapy and bridging therapy. Infections arising in the early phase are more likely related to LD chemo and the expected brief period of grade 3-4 neutropenia. Infections arising in the late phase are particularly worrisome because they are associated with adverse risk features including prolonged neutropenia, dysregulation of humoral and adaptive immunity with lymphopenia, hypogammaglobinemia, and B cell aplasia. Bacterial, respiratory and other viral infections, protozoal and fungal infections can occur during this time . We recommend enhanced supportive care including prompt recognition and treatment of neutropenia with growth factor support, surveillance testing for specific viruses in the appropriate instance, management of hypogammaglobulinemia with repletion as appropriate and extended antimicrobial prophylaxis in those at higher risk (e.g. high dose steroid use and prolonged cytopenia). Finally, we recommend re-immunizing patients post CAR-T based on CDC and transplant guidelines.

## 1. Introduction

Chimeric antigen receptor T-cell (CAR T-cell) is an adoptive immunotherapy utilizing genetically modified autologous T-cells that recognize tumor antigens. This therapy changed the paradigm in the management of refractory or relapsed non-Hodgkin’s lymphoma (NHL), acute lymphoblastic leukemia (ALL), and relapsed refractory multiple myeloma (RRMM). Despite the success, CAR T-cell therapy presents with unique potential toxicity profile, specifically cytokine release syndrome (CRS) and immune effector cell-associated neurotoxicity syndromes (ICANS). While these toxicities prevail in the first few weeks post-infusion, infection is emerging as the most important cause of non-relapse mortality (NRM).

Presently approved CAR T-cell therapies target CD-19, and B cell maturation antigen (BCMA) found on B cells and plasma cells, respectively. CD-19-directed therapies for relapsed and refractory NHL and ALL include axicabtagene autoleucel (axi-cel), tisagenlecleucel (tisacel), brexucabtagene autoleucel (brexu-cel) and lisocabtagene maraleucel (liso-cel).^1–7^ Since 2021, BCMA-directed CAR-T have been approved for RRMM, including idecabtagene vicleucel (ide-cel) and ciltacabtagene autoleucel (cilta-cel).^8,9^ Additional approvals in additional indications and earlier lines of care are anticipated.^1,2^ There is also continued interest in the development of novel CARs, including allogeneic CARs, and in expanding the indications to solid tumors and autoimmune diseases.

Given the morbidity and NRM associated with infections, we review their incidence and patterns and the approach to monitoring and prevention of serious infectious complications of CAR-T therapy. This review describes the general considerations and principles for CD19 and BCMA directed therapies. These principles are applicable in adults and children for the FDA approved indications and may also be useful for investigational CAR-T for NHL and RRMM.

## 2. The emergence of infections as a significant concern

### 2.1. Incidence

The incidence of infection varies for CD19 and BCMA CAR-T recipients. Single center reports demonstrate that the incidence of any grade infections can be over 50% in CD19 CAR-T.^10^ The cumulative incidence of infection with CD19 CAR-T up to 1 year can be 63.3% for axi-cel and tisa-cel, with 23% being severe and most being bacterial.^11^ A single-center retrospective study on axi-cel recipients identified that, in the first month, 36.5% patients developed infection, of which, in 12.9%, was severe.^12^ On univariate analysis, CRS, ICANS, corticosteroids, and tocilizumab use was associated with an increased risk of infection.^12^ Beyond 30 days, Meir et al. reported the incidence of any grade infections/febrile neutropenia in pivotal trials for CD19 CAR-T therapies ranging from 32-43% (any grade) (3-13% grade ≥ 3) for NHL and ALL.^13^ A systematic review of all infections across MM, NHL, and ALL reported a 17% incidence of severe (Grade ≥3) infections, including both early and late infections.^14^ Meanwhile, for BCMA-directed CAR-T, any grade infection was 69% for ide-cel and 58% for cilta-cel (22-20% grade ≥3 respectively).^13^

### 2.2. Mortality due to infectious complications

Infection is emerging as an important preventable cause of NRM. Relapse rates after CAR-T therapy are around 40-60% and disease relapse accounts for the majority of all-cause of deaths in CAR-T recipients. Death from infectious complications accounts for 1% of all-cause deaths and remains the driver of NRM in CAR-T recipients.^10,12,14,15^ When specifically evaluating the causes of NRM, we find that infection is the main driver. A recent investigation on mantle cell lymphoma patients who received brexu-cel reported that NRM was 6% at 180 days, mostly driven by infections.^16^ A recent French DESCAR-T registry study reviewing patients with R/R NHL who received CD19 CAR-T therapy found an overall rate of NRM of 5% in the treated population, most of these occurring beyond Day 30, with infections being responsible for the majority (56%) of NRM events.^17^ Another global report on axi-cel and tisa-cel recipients found that NRM was 5.4% and infections accounted for the highest mortality rate amongst these, at 2.42%.^18^ As for BCMA-directed therapies, the KarMMa trial reported infection-related mortality in 2% of all ide-cel recipients.^8^ The NRM with BCMA therapies in the late period is yet to be described, but is expected to be similar or higher than for CD19 CAR-T, given the increased incidence of infections in BCMA CAR-T recipients.^19^ With the continued use of CAR T-cell therapy as an established treatment in hematological malignancies, and the ongoing early phase studies in solid tumors, it is imperative that we continue to evaluate and strive to understand the infection risk associated with this therapy and develop management strategies with the aim of decreasing NRM.

### 2.3. Phases and patterns of Infections

**Figure 1.**
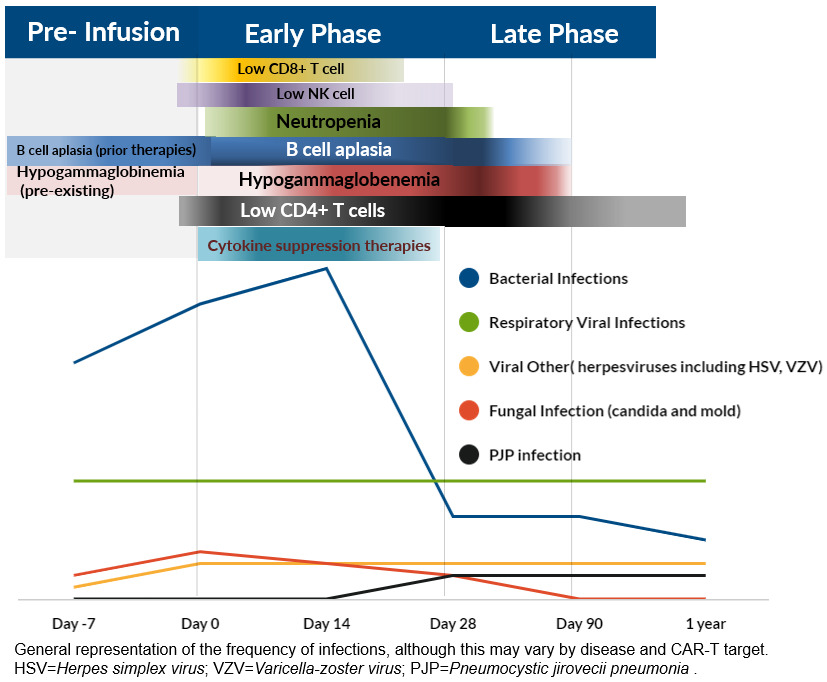
Phases and Relative Frequency: Bacterial, Viral and Fungal Infections

Infections can be broadly divided into periods that are “pre-infusion” ,“early” infections, and “late” infections (Figure 1). The phases are based on the dominant component of the immune state which is compromised and timing from day of cell infusion.^10^

*Pre-Infusion Phase:* This is from time of lymphodepletion (LD) to cell infusion ( Day -7 to Day 0). Patients may have impaired immunity with prior therapies and active disease. Infections during this period are likely attributable to therapy prior to LD (e.g., bridging) and exposures that occurred in proximity to initiation of LD therapy (e.g. respiratory viral infections).

*Early Phase*: This period is from the day of infusion to Day 30, dominated by cytopenia. Due to effects of LD, neutropenia is commonly seen early on during this phase. Lymphopenia and B cell aplasia also develop during this period. CD4+ T cell counts were shown to decrease from baseline and to remain low at a median CD4 of 155cells/mL at 1 year post axi-cel.^20^ CD16/56 NK cells, CD4 and CD8 T cells were all depleted after LD. CD16/56 cells recover typically by Day 28 (14-87 days), and CD 8+ cells by Day 21, while CD4+ cells remain suppressed with inverted CD4/CD8 ratio inversion for up to a year.^21^ While observed with both CD19 and BCMA CAR-T, earlier severe neutropenia, lymphopenia and hypogammaglobulinemia can be seen in BCMA CAR-T compared to CD19 CAR-T recipients.^22^

The presence of high-grade CRS, surge of inflammatory cytokines, and use of immune suppression for management of CRS and neurotoxicity, including tocilizumab and steroids, may impact duration of neutropenia. Infections in the early period are mostly bacterial (68%) and are severe. Moderate viral infections are more prevalent later.^15^

*Late Phase:* The late phase is defined as the period beyond the initial 30 days post-infusion. This phase is dominated by prolonged neutropenia, dysregulation of humoral and adaptive immunity with lymphopenia, hypogammaglobinemia, and B cell aplasia.

Prolonged neutropenia increases the ongoing risk of bacterial infections. Hematological toxicity can have a cumulative 1-year incidence of 58% post-CD19 CAR-T, and may show a biphasic course, with initial neutrophil recovery followed by a “second dip”.^23^ A single center retrospective study of 85 axi-cel patients attempted to characterize the immune reconstitution and infection risk for patients, demonstrating that grade ≥ 3 neutropenia was prevalent in 30% patients at Day 30 and in 7% at 1 year.^20^ Another study demonstrated that, at 2 years, 11% of patients had grade ≥ 3 cytopenia.^24^ BCMA CAR-T recipients had a higher incidence of prolonged neutropenia, with 40% grade ≥3 cytopenia prevalent at Day 90.^13^ Hypogammaglobulinemia onset is typical after 2 weeks post infusion, and occurred in 35%, 27% and 46% of patients between day 15-30, 31-60 and 61-90, respectively.^21,25^ CD19+ B cells have been reported to remain low, resulting in deficiency of the humoral immune system, until a median of 79 days in a study on CD19 CAR-T in ALL patients.^21^ As for BCMA CAR-T recipients, the CAR-T targets BCMA expressing cells, including mature B lymphocytes and plasma cells, which are a vital arm of the humoral immune system.^26^ Moderate viral infections are more prevalent during this period.^15^ Reynold et al. reported that late were as common as early infections in NHL population, while the latter was twofold higher than late infections in ALL.^14^ The patterns of infections differ in these phases and are visualized in Figure 1.

### 2.4. Patterns of infection by organism

*2.4.1. Bacterial infections:* These are common, especially early post infusion. The known real-world incidence and severity is mostly based on retrospective data. In the early CAR-T period, bacterial infections account for 40-50% infections, typically occurring within the first 2 weeks.^11,12,20^ Another study found that up to 90% of bacterial infections occurred in the first 4 weeks with the highest risk with neutropenia.^27^ This includes blood stream infections, central venous catheter-related infections and can be organ specific such as gastrointestinal or respiratory.^11,12,20^ In addition, urinary tract infections were also reported.^28^ Common bacteria include gram positive organisms, gram negative *Enterobacteriaceae*, and *Clostridium*.^11,20^ In a study of ALL children and adolescent CAR-T recipients, the incidence of bacteremia was 39%, with risk factors being IgG ≤ 400 mg/dL, severe CRS and lymphodepleting chemotherapy (LD) other than fludarabine and cyclophosphamide, and prior allogeneic hematopoietic stem cell transplant (alloHCT).^29^

Also, most patients receive empiric broad spectrum antibiotics for neutropenic fever, which can possibly relate to noninfectious causes, such as CRS in the early post infusion period. Such patients are at risk of dysregulation of gut microbiome. A study by Hu et al showed that second- generation CAR-T severe CRS can alter the gut microbiota.^30^ The “microbiome” and efficacy of CAR-T is being studied.^31^

*2.4.2. Viral infections:* One analysis found that 50% of infections in CAR-T recipients were viral, with 80% being respiratory infections.^32^ These are relatively more common after Day 30 for CD19-directed CAR-T. During the first 30 days, they are the second most common after bacterial infections, although they may be higher for RRMM even in the early phase.^28,33^

*2.4.2.1. Non respiratory viral infections:* These include *Herpes simplex virus* (HSV), *Varicella-zoster virus* (VZV), *cytomegalovirus* (CMV) and *Human herpesvirus type 6* (HHV6).^24^ Delayed reactivation of HSV and VZV can be seen around 6-12 months after infusion.^11^ CMV reactivation is seen in 1-2%, although the exact incidence is not known as testing is not frequently done.^34^

*2.4.2.2. Severe Acute Respiratory Syndrome Coronavirus 2 (SARS-CoV-2) infection:* Amongst respiratory viral infections, there has been heightened focus on SARS-CoV-2, which was declared a global pandemic in 2020. CAR-T recipients are one of the highest risk groups for SARS-CoV-2 infection and at highest risk of prolonged viral shedding.^35^ Mortality was around 30-40 % in HCT recipients during the pandemic.^36,37^ A single center report of HCT and CAR-T recipients in 2021 demonstrated that 28% of those with coronavirus disease-19 (COVID-19) infection, had severe disease.^38^ There was prolonged viral shedding for a median of 7.7 weeks .^38^ In another European study of 57 patients, admission for COVID-19 was 80%, with 43% requiring oxygen support and 39% intensive care unit admission.^37^ Over the years, several SARS-CoV-2 variants have emerged, with varying risks of morbidity, including Alpha, Beta, Gamma, Delta and Omicron, with multiple subvariants and sub-branches.^39^ The outcomes vary by variant, and are worse in those patients with lymphopenia, higher ferritin and who develop COVID-19 close to infusion.^38,40,41^ Therapies such as remdesivir, nirmatrelvir/ritonavir and immunizations have contributed to improved outcomes in recent years.^42,43^ Although the World Health Organization (WHO) declared that the pandemic no longer constitutes a ‘public health emergency of international concern’ on May 5, 2023, COVID-19 is still present in localized small outbreaks and is still considered to be a risk for morbidity and mortality in CAR-T recipients.^44^

*2.4.3. Invasive Fungal infections and Pneumocystic jirovecii pneumonia (PJP) :* Invasive fungal infections , including fungal infections occur in 1-5% in CAR-T recipients.^33,45,46^ The risk factors include prolonged neutropenia the use of high dose or long duration of corticosteroids, and severe CRS.^45,46^ This usually occurs within the first month post infusion.^25^ PJP can occur beyond the first month, but overall is rare, likely due to widespread prophylaxis. (Figure 1)

### 2.5. Factors influencing Post CAR-T Infection Risk

Infections occur due to an interplay of several factors, such as immunosuppression with LD and bridging therapies, cytopenia and immune dysregulation with CAR-T, CRS and ICANS.^13^

**Figure 2.**
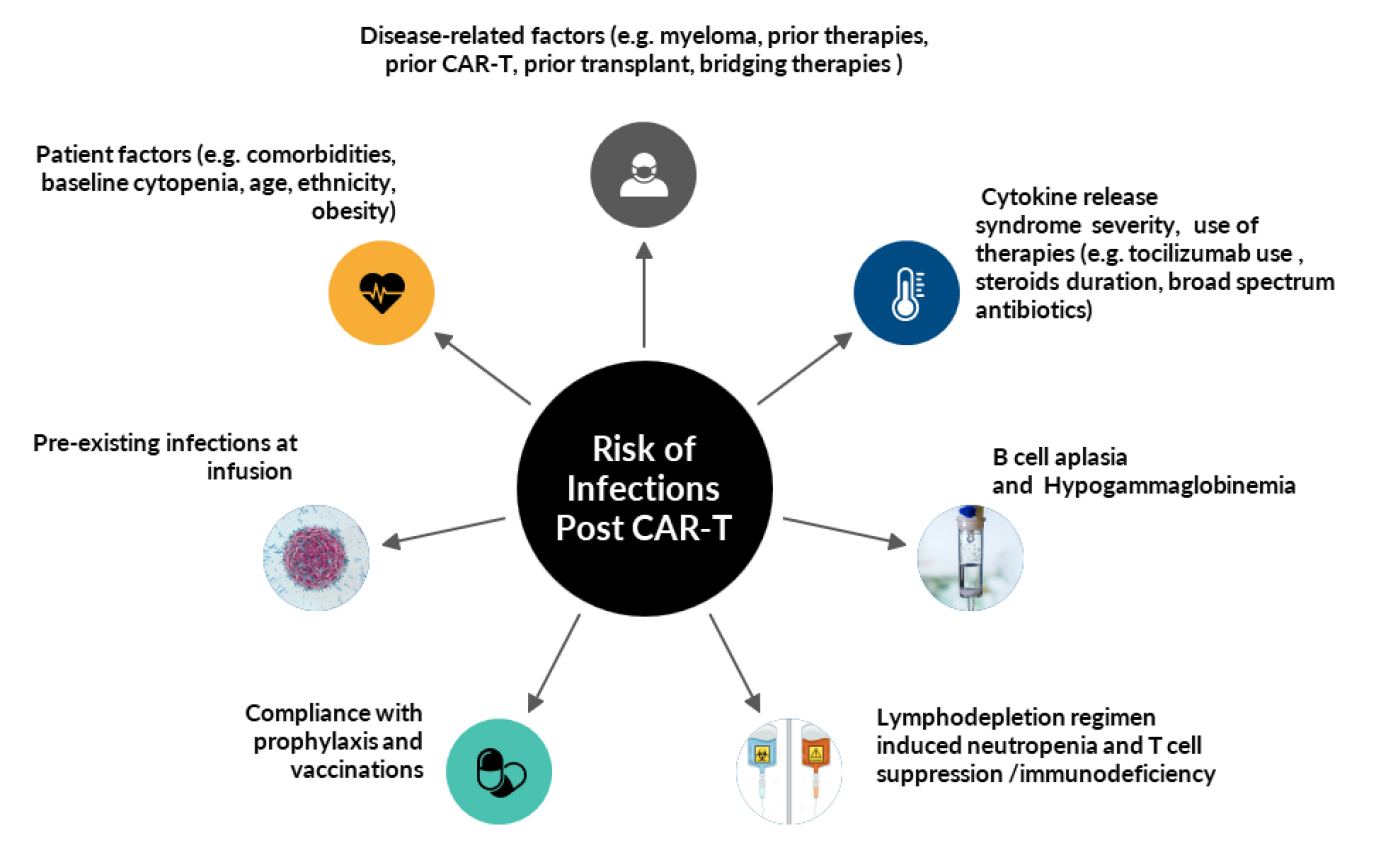
Factors Influencing Post CAR-T Infection Risk

Factors are summarized in Figure 2 and include:

*2.5.1.Patient-Related Factors:* Patient ethnicity, health condition, presence of comorbidities, functional status and chronic infections (e.g. immunodeficiency at infusion, *Human immunodeficiency virus* (HIV) and hepatitis infections).^47^ Jacobson, et al reported that patients receiving axi-cel had broader comorbidities, including 4% with requiring ongoing microbial treatment.^48^ Impaired performance status and history of infections within 30 days before CAR-T was a risk factor for severe bacterial infection.^11^ Post cellular therapy, especially HCT, in patients with COVID-19 infection, age > 50 years was a prognostic factor.^36^ A report by Dayagi et al on 88 patients with relapsed/ refractory leukemia/lymphoma who received CD19 CAR-T demonstrated that children had less severe infections than adults.^49^ Older age is associated with an impairment of the immune system, due to a decrease in immunological diversity of naïve T cells and an increasing number of senescent T cells, which leads to a higher susceptibility to disease and infection.^50^

*2.5.2. Disease-Related Factors:* The risk of any grade infection post CAR-T is highest in RRMM, compared to NHL and ALL patients, with 57% all-grade infections.^14^ RRMM patients also had the highest risk of severe infections including bacterial and viral infections.^14^ IgG subtype and high-grade CRS were risk factors.^22^ Disease or therapy-related cytopenia and lymphopenia at baseline increase the risk of prolonged cytopenia and its associated infections.^24^ Other factors include baseline cytopenia secondary to disease, duration of prior therapies, immunosuppressive therapies previously used (e. g. infusion of CD20 monoclonal antibodies, which is associated with B cell aplasia), prior CAR-T cell therapy and prior alloHCT.^51^ Hypogammaglobulinemia and associated humoral immune dysfunction are additional “on-target, off-tumor” effects related to profound plasma cell aplasia that can last >200 days after cell infusion.^25^ Moreover, lack of response was associated with higher risk of infections.^49^ Maintenance therapies and subsequent therapies, such as BCMA bispecific antibodies for myeloma or CD20 bispecific antibodies for lymphoma also must be taken into consideration when evaluating immune reconstitution and preventative strategies. Further investigation and consensus on management is ongoing.

*2.5.3. LD regimen:* LD is employed to allow effective T cell expansion. The most used regimens include fludarabine and cyclophosphamide (Flu/Cy) or bendamustine. The risk of infection is considered to be higher with Flu/Cy compared to bendamustine.^52^ However, data are conflicting, and another study in ALL patients showed that the risk of infection was highest in non-Flu/Cy LD.^29^ Enhanced LD with higher Flu/Cy doses may also increase the risk of infections.^53^

*2.5.4.CAR-T characteristics:* B cell aplasia is an off-tumor on-target effect of both CD19 and BCMA-directed CAR-T therapies. Novel targets such as mucin-1 targeting with p-MUC-1 or other solid tumor targets that are not expressed on immune cells, may not cause profound immunosuppression. Meanwhile, the impact of BCMA–directed CAR T cells on humoral immune dysfunction may be even more profound than that caused by CD19-directed T cells, because of BCMA expression on long-lived bone marrow plasma cells that produce pathogen–specific antibody responses.^22,54^ The infection risk may vary by co-stimulatory signaling domains (4-1BB versus CD28), which are associated with varying risks of severe CRS.^12^ However, there is no head-to-head comparison between products.

*2.5.5.CRS and ICANS and associated therapies:* CRS and immune dysregulation may lead to tissue damage and endothelial dysfunction, thereby increasing the risk of infections.^13^ Prolonged steroid use with high grade ICANS may predispose CAR-T recipients to developing CMV reactivation and fungal infections. Moreover, febrile neutropenia treated with broad spectrum antibiotics can potentially interfere with the gut microbiome and alter immune reconstitution.

## 3. Infection Prevention

### 3.1. Pre-infusion considerations to reduce risk of infection

#### 3.1.1. Screening prior to leukapheresis and cell infusion

Screening for infections constitutes a pivotal element in the pre-CAR-T-cell therapy risk evaluation.^55^ Active bacteremia , fungal infections and viremia should be controlled prior to collection.^55^ All patients should undergo serologic testing for HIV, *Hepatitis B virus* (HBV), and *Hepatitis C virus* (HCV) within 30 days of leukapheresis.^55–57^ Additionally, serologic screening for HSV and VZV proved valuable in guiding the use of antiviral prophylaxis, specifically with valacyclovir/acyclovir.^56^ This prophylactic measure is recommended throughout the 6 to 12 months post-CAR-T-cell therapy, with potential consideration for future VZV vaccination. Furthermore, CMV serologic screening may be contemplated before CAR-T-cell therapy.^56^ Assessment of latent infections for individual patients, including *Mycobacterium tuberculosis*, human T-cell lymphotropic virus type 1, *Strongyloides stercoralis*, *Toxoplasma gondii* and *Treponema pallidum* can be considered based on their medical history and epidemiologic risk factors.^56,58^ At cell infusion, screening for COVID-19 is recommended to detect asymptomatic infection. Asymptomatic patients testing positive for COVID-19 by qPCR may proceed to CAR-T manufacture, but this is done at risk and at the physician’s discretion.^55^

#### 3.1.2. Active infection at infusion

To minimize the risk of morbidity, patients should not have an active infection at the time of cell infusion.^55^ In those with respiratory symptoms, who should undergo workup to test for the source of respiratory viral illness, a delay should be considered, especially for patients with COVID-19, respiratory syncytial virus, parainfluenza virus 1 to 4, influenza virus A or B, metapneumovirus and adenovirus.^58^ In case of COVID-19 positivity, the delay should ideally be of 14 days, allowing for resolution of symptoms. However, if early infusion is considered, retesting in 5-7 days and proceeding, if testing is negative and the patient is asymptomatic, may be reasonable and warranted, if the potential benefit outweighs the risk.^59^ When possible, cell infusion should be postponed until count recovery from bridging or prior therapy is achieved, to avoid prolonged cytopenia. While factors such as timing of CAR-T, LD chemotherapy and bridging may not be controlled, it is imperative to be aware of these factors and educate patients and monitor for infections.

### 3.2. Prophylactic strategies

These are mostly adopted from consensus statements and practice guidelines^58^ and summarized in Table 1:

*3.2.1 . Antibacterial prophylaxis:* This should be implemented during periods of severe neutropenia, with an absolute neutrophil count (ANC) < 500/mm^3^. Fluoroquinolone prophylaxis is routinely used.^25,58^

*3.2.2.Antiviral prophylaxis:* This is typically prophylactic acyclovir 400mg-800 PO twice a day or valacyclovir 500mg PO twice a day starting at LD and continuing for at least 6 months post infusion.^58^ No prophylaxis for *herpesviruses* other than HSV and VZV is recommended.

*3.2.3.Antifungal prophylaxis:* Antifungal prophylaxis with fluconazole 200mg PO daily is recommended while the ANC is below 500/mm^3^.^16^ Based on the 2018 American Society of Clinical Oncology (ASCO) and Infectious Diseases Society of America (IDSA) guidelines, mold prophylaxis is recommended if the population-level risk of aspergillosis is ≥6%.^60^ While institutional guidelines may vary, mold prophylaxis should, in general, be offered to patients who have prolonged neutropenia (> 3 weeks) , receive high doses of corticosteroids (≥ dexamethasone 10mg per day, for more than 3 days or methylprednisolone ≥ 1gm IV), use of > 1 dose of tocilizumab or second line agents such as anakinra, as well as those with prior mold infections.^46,58^ In these patients, mold prophylaxis for 1 month after completion of immunosuppressive therapy is recommended.^58^

*3.2.3. PJP Prophylaxis*: Trimethoprim/sulfamethoxazole is recommended to be initiated around 1 month post CAR-T, if blood counts allow. Otherwise, other less myelosuppressive options such as inhaled pentamidine, dapsone or atovaquone, should be instituted, typically until the CD4+ count is over 200/uL.^11,61^

*3.2.4.SARS-CoV-2 prophylaxis:* Pre-exposure prophylaxis with neutralizing monoclonal antibodies (tixagevimab/cilgavimab) was recommended in 2021 but is not effective against the new dominant variants and, thus, is no longer recommended presently.^44,62,63^

**Table 1.** Prophylaxis Post CAR-T

**Prophylaxis**	**Preferred Drugs^@^**	**Alternate**	**Start**	**End**
**Antibacterial ( with *Pseudomonas* coverage)**	Fluoroquinolones (Levofloxacin 500mg PO daily)	Cefpodoxime 200mg PO BID	Onset of neutropenia ( ANC < 500/mm^3^)	Recovery of neutropenia ( ANC > 500/mm^3^)
**Antiviral ( for HSV/VZV prevention)**	Acyclovir 400-800mg PO BID	Valacyclovir 500mg PO daily Famciclovir 250mg PO BID	Start with lymphodepletion	Continue until CD4 counts > 200 *or* at least 6 months
**Antifungals**	Fluconazole 200mg PO daily (standard risk) Posaconazole 300mg PO daily	Micafungin 50 IV q24H ( if LFT abnormality)	Onset of neutropenia ( ANC < 500/mm^3^)	Recovery of neutropenia ( ANC > 500/mm^3^)
Antifungals in high risk *	Voriconazole 200mg PO two times a day Posaconazole 300mg PO daily		Start of high dose steroids, other risk factors	Mold prophylaxis for 1 month after completion of IST
**Anti-PJP pneumonia**	Trimethoprim/Sulfamethoxazole 1 double-strength PO three times a week.	Pentamidine 300 mg monthly inhaled. Dapsone 100 mg PO daily. Atovaquone 1500 mg PO daily	Day 28	Continue at least 3 months, until CD4 > 200/uL
***Hepatitis B* carriers/exposed ( HBs Ag positive or Anti-HBc Ab IgG positive)**	Entecavir			At least 6 months and surveillance of LFT and HBV DNA as indicated.

^@^Doses are for adults. Abbreviations: PO: by mouth; BID: twice daily; HSV: ***Herpes simplex Virus***; VZV: ***Varicella zoster Virus***; LD: lymphodepleting chemotherapy; LFTs: Liver function tests, ANC: absolute neutrophil counts; PJP: ***Pneumocystis jiroveci pneumonia***; IST: immunosuppressive therapies * high risk= mold coverage for patients with prior fungal infection, and/or high dose corticosteroids

### 3.3. Immunizations

Vaccination strategies in patients post-CAR T-cell therapy are crucial to ensure the continued protection of these individuals against infectious diseases while considering the unique immunological landscape shaped by CAR T-cell treatment. Immunogenicity and safety of post CAR T-cell vaccinations are unknown, and patients may have a lower response. Extended absence of B-cell function, the depletion of memory B cells, and ensuing hypogammaglobulinemia could compromise the effectiveness of humoral protection against a range of infections, including those preventable through vaccination.^64^ A thoughtful and tailored approach to vaccination becomes imperative to strike a balance between promoting immune response and preventing potential complications.^58^ Furthermore, a personalized and comprehensive assessment of each patient’s vaccination status and history is crucial for guiding vaccination decisions. This includes evaluating prior immunizations, assessing the need for booster shots, and considering any pre-existing immunity.

One key aspect of vaccination strategies in post-CAR T-cell therapy patients involves careful timing. Vaccination should be deferred until the recovery of immune function, which may take several weeks to months after CAR T-cell infusion. This delay allows the immune system to regain its strength, reducing the risk of adverse events and ensuring the effectiveness of the vaccines. Additionally, prioritizing vaccinations against vaccine-preventable diseases, such as influenza, pneumococcus, and other common pathogens, is essential. Moreover, the choice of vaccines may need to be adjusted based on the specific nature of the patient’s underlying malignancy and the potential impact of the CAR T-cell therapy on the immune response.

*3.3.1.Respiratory viral vaccinations:* The recommendations are summarized in **Table 2.**

**Table 2.** Vaccinations for Respiratory Viruses

**Respiratory Virus**	**Pre-CART**	**Post-CART**	**Comments**
**Influenza**	2 weeks prior to LD	1- 3 mo. post CART irrespective of immunological reconstitution	Low likelihood of serological response in B cell aplasia. Consensus view is that vaccination may still be beneficial to reduce rates of infection and improve clinical course. Consider boost upon B-cell recovery^65^
COVID-19*	Primary series complete at least 2 weeks prior to LD	>3 mo. after CAR-T cell infusion	Relies heavily on T-cell-mediated immunity, therefore B-cell aplasia does not seem to be a contraindication; no T-cell threshold has been defined. Guidance on re-vaccination post-CAR-T and frequency/dosing of booster vaccines will vary between countries. National guidelines should be followed
**RSV**		> 3 mo. post infusion	Approved in 2023 for adults > 60 yr old.

COVID-19= coronavirus disease-19; RSV=respiratory syncytial virus

The influenza vaccine should be given annually prior to the expected influenza season, and 2 weeks prior to LD chemotherapy. The introduction of mRNA vaccines has resulted in favorable response rates for SARS-CoV-2, even with vaccination as early as 3 months post-HCT.^66^ This development provides a compelling basis for contemplating their use in recipients of CAR-T-cell therapy. The RSV vaccination was recently approved in 2023 for all adults ≥ 60 years old.^67^ Based on our expert opinion, the RSV vaccination can be offered post Day 100.

*3.3.2. Inactivated vaccinations:* Consensus guidelines recommend the administration of killed or inactivated vaccines at least three months after CAR-T therapy.^44,64^ Before vaccination, patients should exhibit immune reconstitution, characterized by CD4+ counts exceeding 0.2×10^9^/L and CD19 or CD20+ B cell counts surpassing 0.2×10^9^/L. It is crucial that patients have not undergone concurrent immunosuppressive treatments, including cytotoxic chemotherapy, systemic corticosteroids, T-cell-depleting or anti-lymphocyte agents, or intravenous immunoglobulin (IVIg) within the preceding two months.^64^

*3.3.3. Live Vaccinations:* Divergent guidelines advise maintaining a distinction in the administration of live vaccines until at least one-year post-CAR-T therapy, contingent upon the demonstration of immune reconstitution. Some expert groups go further to contraindicate live vaccines within the initial eight months following intravenous IVIg replacement.^65^ Notably, centers such as the Fred Hutchinson Cancer Center recommend a delay in both live and non-live adjuvant vaccines, until a minimum of five months have elapsed since the last IVIg replacement.^68^ Ultimately, a well-considered vaccination strategy is integral to promoting long-term immune health in individuals who have undergone CAR T-cell therapy, reducing the risk of infectious complications and supporting their journey towards sustained remission.

**Figure 3.**
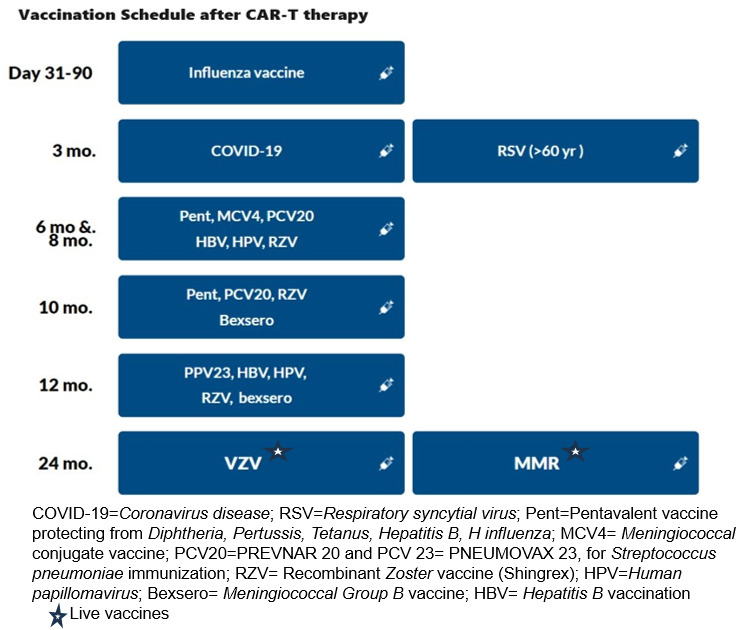
Vaccination Schedule after CAR-T therapy

The recommendations for vaccinations after CD19 and BCMA CAR-T, based on the European Society for Blood and Marrow Transplantation ( EBMT), the Joint Accreditation Committee of ISCT and EBMT (JACIE), and the European Hematology Association ( EHA) consensus are shown in **Table 3 and Figure 3** .^65^ It is important to note that most of these strategies are largely based on expert opinion, extrapolated from autologous transplant protocols.^25,55^

**Table 3.** Considerations for Immunizations for inactivated and live vaccines in adults (excluding respiratory virus vaccinations) .

**Factor**	**Comment**
**Prolonged neutropenia/thrombocytopenia**	Beyond 6 months, if prolonged cytopenia (severe neutropenia/thrombocytopenia) despite supportive care, would consider delaying vaccinations. There is no consensus on such scenarios.
**B cell aplasia**	Increases risk of encapsulated organisms such as Haemophilus influenzae type B, Neisseria meningitidis, streptococcus pneumoniae. CD19 negative plasma cells may play a role in developing humoral immunity to this vaccine preventable infections.
**Timing post infusion**	≥ 6 months post CAR-T for inactive vaccines and at least ≥ 12 months post infusion for live vaccinations, except influenza and COVID vaccinations which are offered earlier, to mitigate risk of getting the infections.
**Immunoglobulin (IVIg) infusions**	Non respiratory virus: Inactivated Vaccinations are offered at least 2 months after IVIg replacement . Live Vaccines are typically given > 8 months after IVIg.
**Use of ongoing cytotoxic therapies or immunosuppressives**	Non respiratory virus: Inactivated Vaccinations : Recommend avoiding the use of inactivated vaccinations if ongoing immunosuppression. Recommendations are evolving on patients who relapse and may be on bispecific agents, or other novel therapies. Live vaccinations : Recommend to avoided in patients on active chemotherapy, except for therapies that do not actively suppress B and T cell function such as checkpoint inhibitors, immunomodulatory agents (e.g. lenalidomide) , tyrosine kinase inhibitors, and other select agents.^58^
**Absolute CD4 count 0.2×10^9^/L**	This element of immune reconstitution is an important consideration for live vaccinations.
**Prior allogeneic or autologous hematopoietic stem cell transplant (HCT)**	Vaccination recommendations in CAR-T patients who previously received an HCT and re-immunizations may be guided by serum antibody testing, although there is lack of data in this population.^58^ Live vaccines are only to be administered ≥ 2 years from autologous or allogeneic HCT.

### 3.4. Monitoring and Supportive Care

The management of infections is largely based on expert opinion and extrapolation from transplant protocols. There are no recommended surveillance strategies beyond neutropenic fever management for bacterial infections.

*3.4.1. Respiratory Viral Infection Monitoring*: For respiratory infection, it is recommended to check a respiratory viral panel only if symptomatic or exposed. There is no routine serological testing recommended, but this would be considered in targeted settings.^58^

*3.4.2. CMV monitoring:* In high- risk patients, those with receiving significant immunosuppressive treatment for management of CRS and ICANS, weekly CMV monitoring for up to 1 month after discontinuation of therapy is recommended (Table 4).^16^

### 3.5. Treatment of Active Infection

Prompt evaluation and management are crucial, particularly for neutropenic patients in the early post-infusion phase. The treatment of infections in patients post CAR T-cell therapy requires a nuanced and vigilant approach, due to the altered immune landscape resulting from this advanced form of cancer treatment. This should include a multidisciplinary team involving infectious disease specialists, hematologists and other healthcare providers, to ensure comprehensive and timely management of potential infections. Prompt identification and targeted treatment of infections are paramount, given the heightened susceptibility of these patients to opportunistic pathogens. Antimicrobial therapy in post-CAR T-cell therapy patients often involves a judicious selection of antibiotics, antifungals or antivirals, based on the pathogens and the patient’s clinical presentation. The choice of antimicrobials may need to be tailored to the individual’s immunization history, local epidemiology, and the potential for drug interactions with ongoing CAR T-cell-related medications. Additionally, close monitoring for signs and symptoms of infection plays a crucial role in early detection and intervention.

The most important strategy to prevent infection-related deaths, especially beyond the early phase, is to educate referring physicians, patients and caregivers about the morbidity and mortality associated with infectious complications. Instructions on preventative measures such as social distancing, hand washing, immunizations and adherence to prophylaxis are crucial. Furthermore, the education of patients, caregivers, and their local physicians about the immune dysregulation with CAR-T, the need for prompt evaluation of respiratory symptoms, fever or other symptoms is critically important. Recognizing that infections are the main cause of NRM and can occur not only in the immediate post-infusion period but also in the later phase, highlights the necessity for a sustained focus on its prevention. Building an infrastructure beyond 30 days, where immunocompromised patients, such as CAR-T recipients, may be able to bypass the emergency department, where long wait times are expected in a space shared with other patients who may have respiratory viruses, may help reduce their exposure to other infections. Given the risk of opportunistic infections in this population, it is imperative that there be a mechanism for direct written and oral communication between the authorized treatment center (ATC) physicians and referral physicians, as well as availability of the ATC team 24/7 for referral team guidance.

**Table 4.** Supportive Care

**Immune Reconstitution Arm**	**Strategies**	**Monitoring**	**Comments**
**Neutropenia**	GCSF (filgrastim or biosimilar) support	CBC every 1-3 days in first 4 weeks, then weekly or as indicated.	Use if ANC < 1000/mm^3^ or if ANC has not recovered to >500/mm^3^ by day 7 to 10 Avoid the use of GCSF in patients with ongoing CRS, unless there is concomitant serve infection^69^
**Prolonged neutropenia ( > 28 days)**	TPO agonists^69,70^ (eltrombopag) 75 mg daily with consideration to increase to 150 mg	Atleast weekly or more frequently if indicated	Stop TPO agonists once platelets recover or if no response observed in 4-6 weeks after initiation^69^
	CD34 Stem Cell Boost		Consider if stored stem cells ( myeloma)^71^
**Hypogammaglobulinemia, B cell aplasia**	Immunoglobulin infusion	IgG levels monthly	Replace when IgG <400 mg/dL
**Lymphopenia**	No supportive care	Lymphocyte subset / CD4 counts monthly	Used to determine when to stop antiviral and PJP prophylaxis. (CD4 > 200 /µl)
**Significant Immunosuppressive therapies: for CRS/ICANS (> 1 dose tocilizumab, ≥ dexamethasone 10mg daily for 3 days or methylprednisolone 1gm IV, or anakinra/second lne agent use)^72^**	Consider mold prophylaxis until 1 month after completion	Weekly CMV monitoring	

GCSF= granulocyte colony stimulating factor; CRS=cytokine release syndrome; TPO=thrombopoietin ; CMV=cytomegalovirus; IgG=immunoglobulin; PJP= ***Pneumocystis jirovecii pneumonia***

### 3.6. Supportive Care

Supportive strategies play a pivotal role in managing infections in post-CAR T-cell therapy patients (Table 4). Enhancing the patient’s immune system and managing cytopenia can contribute significantly to reducing the risk of serious infections.

*3.6.1: Neutropenia:* Evaluation of the etiology of prolonged neutropenia is important. Supportive care such as granulocyte-colony stimulating factor (GCSF) may be considered to boost neutrophil counts and enhance the body’s ability to fight infections.^69,73^ The thrombopoietin-receptor (TPO) agonist eltrombopag, is also used for prolonged neutropenia, given retrospective evidence of trilineage hematopoietic improvement in the post- CAR-T setting.^28,70,74^ CD34+ stem cell boost can also shorten the time to neutrophil recovery.^71^

*3.6.2. Hypogammaglobenemia:* There is a consensus that IgG levels should be monitored before CAR-T-cell therapy infusion and monthly, for at least three months afterwards.^56,57,65^ The benefits of universal prophylaxis with IVIg replacement therapy (IGRT) in the context of asymptomatic hypogammaglobulinemia remain unclear. Most of the data on IGRT are extrapolated from patients who receive CD20 monoclonal antibody and alloHCT recipients.^10,75^ Criteria that might be particularly relevant for prompt initiation of IGRT include the presence of serious or recurrent bacterial infections when the total serum IgG level is below 400 mg/dL or per institutional protocol.^72^ Notably, recipients of BCMA CAR-T-cell therapy exhibit more profound humoral deficits, suggesting a potentially higher benefit from a more liberal use of IGRT. However, to identify patients who would derive the most benefit, as well as to determine the optimal timing, schedule, duration, and modality of IGRT, prospective controlled trials are essential.^75^

## 4. Future Directions and Research

Understanding the implications for the future of CAR-T therapy and patient outcomes is vital in shaping effective infection management strategies.

Further research is needed to better understand immune reconstitution and to develop strategies to minimize or manage prolonged cytopenias. There is an unmet need to identify modifiable baseline risk factors, define neutropenic fever in the context of cytokine release syndrome, determine the benefit of anti-mold prophylaxis, and establish evidence-based approaches to antifungal prophylaxis and diagnosing the risk of life-threatening infections using cytokine patterns. Additionally, more research is warranted to understand the factors contributing to recurrent viral infections, the role of different LD regimens and intensities in defining the patterns of infection, and to develop novel targets and constructs with lower risk of adverse toxicities. Finally, educational material and standard transition of care guidelines for referring oncologists, with an outline on monitoring and surveillance for infections is important to identify and treat infections promptly.

## 5. Conclusions

In conclusion, the key findings underscore the critical role of prophylactic and preventative strategies, early recognition, and treatment of infection since this is the primary driver of NRM in CAR-T recipients. Prophylactic and preventative strategies for all patients are imperative to mitigate the risk of serious infections. Furthermore, the importance of supportive strategies for immune reconstitution and effective cytopenia management is emerging as a pivotal aspect of patient care, potentially contributing to a reduction in infection-related complications. The time-sensitive nature of prompt evaluation and management, particularly in the early post-infusion period for neutropenic patients, is a crucial element in enhancing patient outcomes. The broader implications for the future of CAR-T therapy and patient well-being extend beyond the initial four weeks, highlighting the need for ongoing vigilance and a sustained focus on infection prevention, even after patients have returned to the community. Improved communication with the ATC beyond the early phase will be important to guide infection monitoring and treatment. This comprehensive understanding of infection dynamics in CAR-T recipients serves as a foundation for refining therapeutic approaches and optimizing patient care to enhance the overall success of CAR-T therapy.

### Author contributions

Conceptualization: Nausheen Ahmed (Equal), Olalekan Oluwole (Equal), Zahra Mahmoudjafari (Equal), Nahid Suleman (Equal), Joseph P McGuirk (Equal). Writing – original draft: Nausheen Ahmed (Equal), Zahra Mahmoudjafari (Equal). Writing – review & editing: Nausheen Ahmed (Equal), Olalekan Oluwole (Equal), Zahra Mahmoudjafari (Equal), Nahid Suleman (Equal), Joseph P McGuirk (Equal).

### Competing Interests

NA: Consultancy: Kite/Gilead; Advisory Board: Bristol-Myers Squibb.

OO: Consultancy: Pfizer, Kite, Gilead, AbbVie, Janssen, TGR therapeutics, Bioheng, ADC, Novartis, Allogene, Nektar, Caribou.

ZM: Advisory board participation: KITE, BMS, Genentech, Janssen, Pfizer

JPM: Consultant for: Kite, Allovir, Novartis, Nektar, Bristol Myers Squib, Envision,

Sana, Legend Biotech, CRISPER, Autolus

NS reports no conflict of interest
